# Get Tough, Get Toxic, or Get a Bodyguard: Identifying Candidate Traits Conferring Belowground Resistance to Herbivores in Grasses

**DOI:** 10.3389/fpls.2016.01925

**Published:** 2017-01-05

**Authors:** Ben D. Moore, Scott N. Johnson

**Affiliations:** Hawkesbury Institute for the Environment, Western Sydney UniversityRichmond, NSW, Australia

**Keywords:** root defense, Poaceae, plant secondary metabolites, silica, physical defence, benzoxazinoids, indirect defence, root herbivory

## Abstract

Grasses (Poaceae) are the fifth-largest plant family by species and their uses for crops, forage, fiber, and fuel make them the most economically important. In grasslands, which broadly-defined cover 40% of the Earth's terrestrial surface outside of Greenland and Antarctica, 40–60% of net primary productivity and 70–98% of invertebrate biomass occurs belowground, providing extensive scope for interactions between roots and rhizosphere invertebrates. Grasses invest 50–70% of fixed carbon into root construction, which suggests roots are high value tissues that should be defended from herbivores, but we know relatively little about such defenses. In this article, we identify candidate grass root defenses, including physical (tough) and chemical (toxic) resistance traits, together with indirect defenses involving recruitment of root herbivores' natural enemies. We draw on relevant literature to establish whether these defenses are present in grasses, and specifically in grass roots, and which herbivores of grasses are affected by these defenses. Physical defenses could include structural macro-molecules such as lignin, cellulose, suberin, and callose in addition to silica and calcium oxalate. Root hairs and rhizosheaths, a structural adaptation unique to grasses, might also play defensive roles. To date, only lignin and silica have been shown to negatively affect root herbivores. In terms of chemical resistance traits, nitrate, oxalic acid, terpenoids, alkaloids, amino acids, cyanogenic glycosides, benzoxazinoids, phenolics, and proteinase inhibitors have the potential to negatively affect grass root herbivores. Several good examples demonstrate the existence of indirect defenses in grass roots, including maize, which can recruit entomopathogenic nematodes (EPNs) via emission of (E)-β-caryophyllene, and similar defenses are likely to be common. In producing this review, we aimed to equip researchers with candidate root defenses for further research.

## Introduction

Grasses (the family Poaceae) evolved 66 million years ago (Piperno and Sues, 2005) and have been exploited by humans for around 12,500 years (Baker, 2009). In fact, just three grass species (wheat, rice, and maize) provide 50% of the World's food (Varshney et al., 2012) and other species are important sources of forage, fuel, and fiber (Blair et al., 2014). Grasslands also represent crucial ecosystems, storing up to a third of global climate stocks and account for up to 40% of terrestrial land mass (Gibson, 2009). In grasslands, between 40 and 60% of net primary productivity occurs belowground (Coleman, 1976) and between 70 and 98% of invertebrate biomass is located in the soil (Curry, 1994). Interactions between grass roots and invertebrates must therefore be extensive, yet there are key gaps in our knowledge about these interactions, particularly in terms of plant defenses and root herbivory. Here, we identify candidate grass root traits that assist in resisting herbivory, including physical and chemical defenses and indirect defenses (i.e., herbivore natural enemy recruitment). Where information is available, we describe the efficacy of defenses (sometimes in above-ground tissues or against vertebrate herbivores where that is the only information available), their occurrence in grasses and their documented or likely occurrence in grass roots. It should be noted that in plants generally, including grasses, secondary metabolites found in aboveground tissues of plants are commonly also found in their roots (Rasmann and Agrawal, 2008).

## Herbivores of grass roots

Apart from a few mammals, such as pocket gophers, grass roots are principally attacked by plant parasitic nematodes and herbivorous insects (Andersen, 1987). Plant parasitic nematodes can consume as much net primary productivity as do cattle, and are probably the biggest single group of root feeders in grasses (Seastedt and Murray, 2008). Nematode herbivores are ubiquitous root feeders in grasslands, whereas insect herbivores appear to show particular geographical distributions. In North America and Australasia, scarab larvae are regarded as the most important belowground herbivores in grasslands (Seastedt and Murray, 2008; Frew et al., 2016a), whereas leatherjackets (Tipulidae) and wireworms (Elateridae) are the dominant root-feeding insects in European grasslands (Blackshaw and Kerry, 2008; Seastedt and Murray, 2008). Less well-recognized is the ability of Collembola to act as root herbivores under some circumstances (Endlweber et al., 2009).

While root herbivores are undoubtedly less diverse than shoot herbivores (Johnson et al., 2016b), up to 21 insect species may feed on a the roots of a single plant species (van Dam, 2009) and they show varying degrees of host specificity (Van Der Putten, 2003). With specialists more common in agricultural (Van Der Putten, 2003) and natural (Van der Putten and Van der Stoel, 1998) grass monocultures. Western corn root-worm (*Diabrotica virgifera virgifera*) is a highly specialized feeder on maize (*Zea mays*) and its historical relatives (e.g., teosinte) and has evolved counteradaptations to that plant's root defenses (Robert et al., 2012). African black beetle (*Heteronychus arator*) is a generalist, feeding on more than 190 species of grasses from 33 genera in Australia alone (Hangay and Zborowski, 2010) as well as numerous other monocots and dicots (Frew et al., 2016a). Many grass root herbivores readily switch between hosts, illustrated by the grayback canegrub (*Dermolepida albohirtum*) which was originally a feeder of Australian native grasses, but switched grass species to become a highly destructive pest of sugarcane when it began to be cultivated in Queensland in the early twentieth century (Allsopp, 2010; Frew et al., 2016a). The economic status of these particular grass root feeders has most likely biased research efforts toward certain groups, especially those that chew roots and potentially induce different types of defensive response than fluid-feeding groups. The latter do exist, however, and include sporadically damaging pests such as the mealy grass root aphid (*Aploneura lentisci*), rice root aphid (*Rhopalosiphum rufiabdominale*), and pasture mealybug (*Balanococcus poaea*). Most of our knowledge about grass root defenses, however, appears to come from attack by chewing root herbivores.

Even minor root herbivory can damage plants and alter their physiology by (i) decreasing nutrient and water uptake, (ii) causing disproportionate resource losses by severing roots, (iii) diverting assimilates away from shoot growth for root re-growth belowground, (iv) imposing leaf water deficits, and (v) aggravating pathogen infection (Zvereva and Kozlov, 2006; Johnson and Murray, 2008). From a global agricultural perspective, root herbivores are amongst the most economically damaging, persistent and difficult to detect and control (Johnson et al., 2016a).

## Why would grasses defend their roots?

Plant defense can be divided into two main strategies, tolerance of, and resistance to herbivory (Strauss and Agrawal, 1999), and plants often invest in both of these strategies simultaneously (Núñez-Farfán et al., 2007). Grasses commonly invest 50–70% of fixed C in root construction (De Deyn et al., 2003) and as roots are essential for water and nutrient uptake, it seems likely that grasses defend them (Rasmann and Agrawal, 2008; van Dam, 2009). Most grasses have adventitious, dense root systems with many fine, fibrous axes (Ciamporová et al., 1998), and relatively low nitrogen (N) concentrations compared with forb roots (Tjoelker et al., 2005), although roots can contain significant starch reserves. Generally poor nutritional quality may sometimes lower the risk of herbivory and reduce the need for explicit defense. Plants generally are less tolerant of root herbivory than of shoot herbivory (Zvereva and Kozlov, 2012), although tolerance remains an important component of belowground defense (Rasmann et al., 2011) and herbaceous plants may be better able to compensate for root herbivory than woody plants (Massad, 2013). Chemical defense of roots is also common (van Dam, 2009), although relative concentrations of defense compounds found in above- and below-ground plant parts varies among plants (Rasmann and Agrawal, 2008).

Because so little is known of below-ground defense in grasses, it is worthwhile considering what is known of above-ground grass parts. Many grasses are extremely tolerant of herbivory, particularly when abundant resources are available for regrowth (McNaughton, 1979; Hamilton et al., 1998; Hawkes and Sullivan, 2001), mostly because their growth and regrowth occurs at basal intercalary meristems that are protected by hard leaf sheaths that allow regrowth after herbivory to occur almost immediately (Haukioja and Koricheva, 2000). As a consequence of this architecture and the usual lack of abscission of grass leaves, grasses often show overcompensatory above-ground growth and overcompensatory photosynthesis after above-ground herbivory as the sward is reduced and more light reaches the meristems (Alward and Joern, 1993; Rosenthal and Kotanen, 1994). In contrast, simulated defoliation of trees often reduces growth (Heichel and Turner, 1984), although a recent metanalysis emphasized the diversity of responses within growth forms (Massad, 2013). Although grasses produce roots with apical meristems at the root tip, they differ from most eudicots in developing roots from multiple sites above and belowground (Sebastian et al., 2016) and this greater degree of modularization may also limit damage from root herbivory and facilitate compensatory growth.

The most obvious aboveground herbivore-resistance traits of grasses are physical, and include the deposition of silica phytoliths (Hartley and DeGabriel, 2016) and high proportions of cellulose and lignin, while chemical resistance traits are generally viewed as less significant (with notable exceptions, e.g., Vicari and Bazely, 1993). However, the apparent general lack of chemical defense in grasses may reflect a lack of investigation and a focus on a few economically important species (Kellogg, 2015). This knowledge gap is magnified still further when attention is turned to grass roots, as these are almost always ignored, even when aboveground defenses are investigated.

Investment in resistance traits can require resources that plants could otherwise direct toward growth and reproduction and thus, optimal defense theory (ODT) predicts that allocation of resources to these traits will be driven by the relative costs and benefits of this investment. Investment costs are influenced by the biosynthetic cost and composition of chemical defense, as well as the opportunity cost of forgone growth and reproduction, and the benefits of investment are determined by the vulnerability of plants and plant parts to herbivory and the value of these plant parts to the plant (Zangerl and Rutledge, 1996). The application of ODT to roots lags well-behind its application to above-ground plant parts, largely due to difficulties in determining these costs, values, and degrees of vulnerability (van Dam, 2009). If ODT is applied simply to the question of whether grasses should invest more in the root or shoot resistance to herbivores, however, the apparent reliance on tolerance over resistance for above-ground defense and the comparatively lower tolerance of roots combined with their value to the plant, suggests that chemical defense may be at least, if not more, important for roots.

If herbivore attack is rare or unpredictable, plants can often defer and potentially avoid defense costs by inducing defenses in response to herbivory (Karban and Baldwin, 1997), and this strategy is likely to be as common below-ground as above (Rasmann and Agrawal, 2008). This strategy is observed in all three types of defense discussed below.

## Get tough—physical defenses

Physical defense is a first line of defense against herbivores (Hanley et al., 2007), and in shrub and tree leaves can explain more variation in chewing herbivory than chemical defense (Caldwell et al., 2016). It can prevent or discourage attack by chewing and piercing herbivores, and make nutrients inaccessible or indigestible. Obvious physical defenses such as thorns and trichomes do not occur belowground, though root hairs are the developmental equivalent of leaf trichomes and the product of neofunctionalization arising from a gene duplication event (Kellogg, 2001). There has been some speculation that root hairs may offer some protection by preventing very small herbivores (e.g., neonates) from reaching and penetrating the root epidermis or may possibly provide refugia for natural enemies of herbivores such as entomopathogenic nematodes (Johnson et al., 2016a). Although not strictly a physical defense, root hairs may also increase the root surface available for colonization by beneficial soil microbes, which in turn can sometimes confer resistance to insect and nematode herbivores of grasses (Piskiewicz et al., 2009; Santos et al., 2014). Notably, root hairs can be induced, for example by plant-parasitic nematodes in barley (Haase et al., 2007).

More important belowground, both for defense against root herbivores and for protection against inadvertent uprooting by grazing ungulates, is the resistance of roots to shearing, puncturing, and tearing. This is a product of the architecture and physico-chemical composition of roots. Crystalline deposits of silica and calcium oxalate may play important roles (discussed below), but variation in the proportion of cortex and stele, and corresponding differences in cellulose, lignin, callose, and suberin composition all contribute to root toughness (Gregory, 2006), for example the strength of turfgrass rhizomes and stolons can be best explained by lignin concentrations (Lulli et al., 2011). To date, general patterns of structural chemical composition that confer greater root strength have not been sought or identified, and we suggest that this would be a worthwhile research aim.

The roots of annual and perennial grasses also differ in key attributes including specific root length, root tissue density, modal root diameter and root nitrogen concentration (Roumet et al., 2006), traits associated with nutrient and water acquisitiveness, root lifespan, and relative growth rate (Perez-Harguindeguy et al., 2013). Differences in specific root length can result from either low tissue density or low diameter (Perez-Harguindeguy et al., 2013), with thin roots exerting less penetrative force on soil and transporting less water, and denser roots showing longer longevity. In leaves, toughness per density makes a greater contribution to mechanical strength than lamina thickness and tissue density combined (Onoda et al., 2011), and the same may be true for root diameter and density. In both grasses and trees, the tensile strength of roots decreases with root diameter (i.e., thinner roots are stronger) and this can be explained partly by a scaling effect commonly seen in fracture mechanics and partly by the higher cellulose concentrations observed in fine roots (Genet et al., 2005; Teerawattanasuk et al., 2014).

To our knowledge, only one study has directly investigated the effects of root toughness on root herbivory (Johnson et al., 2010). It found a positive correlation between fracture toughness and root penetration time by *Agriotes* spp. wireworms (Coleoptera: Elateridae), mediated by lignin concentration and composition, suggesting that root toughness could be an effective barrier to root herbivory.

Many, if not most, grasses form rhizosheaths along much of their root length (Goodchild and Myers, 1987; Kellogg, 2015). This casing comprises mineral earth, root hairs and living cap cells, held together by mucilage and is especially well-developed in mesophytic and xerophytic grasses (McCully, 1995, 2005). Particularly when allowed to dry, the rhizosheath forms an integral part of the root, to which it adheres firmly and shows a degree of strength when excavated (Watt et al., 1994). Furthermore, the distribution of soil particle sizes in rhizosheaths is shifted significantly toward smaller particles, relative to the surrounding soil (Ma et al., 2011). As the movement of both nematode and insect herbivores is substantially retarded by increasing soil density (Johnson et al., 2004; Barnett and Johnson, 2013), it may be possible that rhizosheaths afford some degree of protection from root herbivores.

### Silica

In grasses, a major component of physical resistance to aboveground herbivory is via deposition of silica (SiO_2_), a defense that, unusually, may be used more extensively by grasses than by other plants (Hodson et al., 2005). Silica has been linked to drought resistance, structural strength, disease resistance and defense against a range of insect herbivores, the latter via reductions in digestibility and mouthpart wear (Hartley and DeGabriel, 2016). Silica is taken up by roots in the form of monosilicic acid, before being transported to the site of concentration and deposition. There it polymerises as opaline silica, either as a varnish or as morphologically-diverse phytoliths. In many grass species, silica deposition in grass leaves and stems is induced by above-ground herbivory, particularly by vertebrates (Hartley and DeGabriel, 2016), and the same above-ground response was seen in two grasses after root herbivory by scarab beetle larvae (Power et al., 2016), although root silica was not measured in that study.

**Figure 1:**
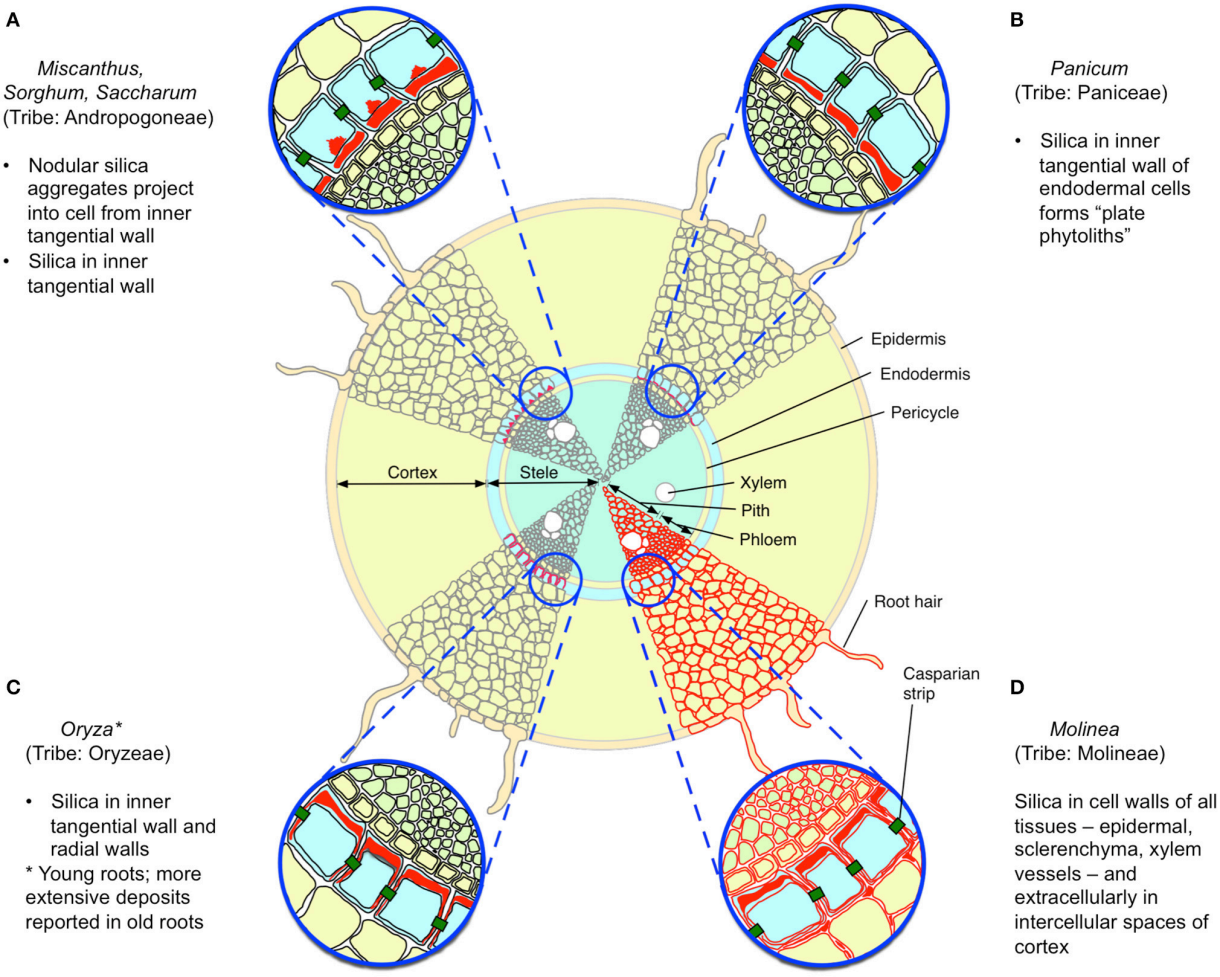
**Reported patterns of silica deposition in grass roots**. Four patterns are illustrated, with silica indicated in red, based upon descriptions from Sangster (1978) and Parry et al. (1984). **(A–C)** Silica is deposited in the endodermis, particularly in the inner tangential wall; in **(A)**, nodular aggregates are also observed inside the cell. **(D)** Silica is found in the cell walls of all root tissues, and extracellularly in the root cortex. Other reported patterns include deposition in all endodermal cell walls (e.g., some wheat and barley cultivars) and no apparent deposition (e.g., *Phragmites australis*).

Silica was first reported from sorghum roots in 1924 (Parry and Kelso, 1975) and its distribution in roots has subsequently been described for several species (Table 1). Total concentrations of silica in wild grass roots can sometimes substantially exceed those observed aboveground (McNaughton et al., 1985; Seastedt et al., 1989) but this varies among species; for example the roots of *Phragmites* possess negligible silica, despite its abundance above ground (Schaller et al., 2013) whilst the thick, long-lived cord roots of *Molinia* (also from the tribe Molinieae in the subfamily Arundinoideae) deposits extracellular silica in all root tissues including epidermal, schlerenchyma, and xylem vessels, forming an almost complete cylinder (Parry and Kelso, 1975). Although the anatomical distribution of silica in roots has only been described in detail for a few grasses (Figure 1), mostly crops, the most common pattern among those species is of deposition on the inner transverse cell walls (and sometimes more extensively) of the endoderm (Parry et al., 1984). This pattern does not seem to be ideal for defense of the root cortex, even though most root nutrients, particularly stored carbohydrates, are to be found there. More in line with the predictions of the ODH, several studies have reported greater silica concentrations in basal proximal) than in apical (distal) roots (Parry and Kelso, 1975; Hodson and Sangster, 1989).

**Table 1:**
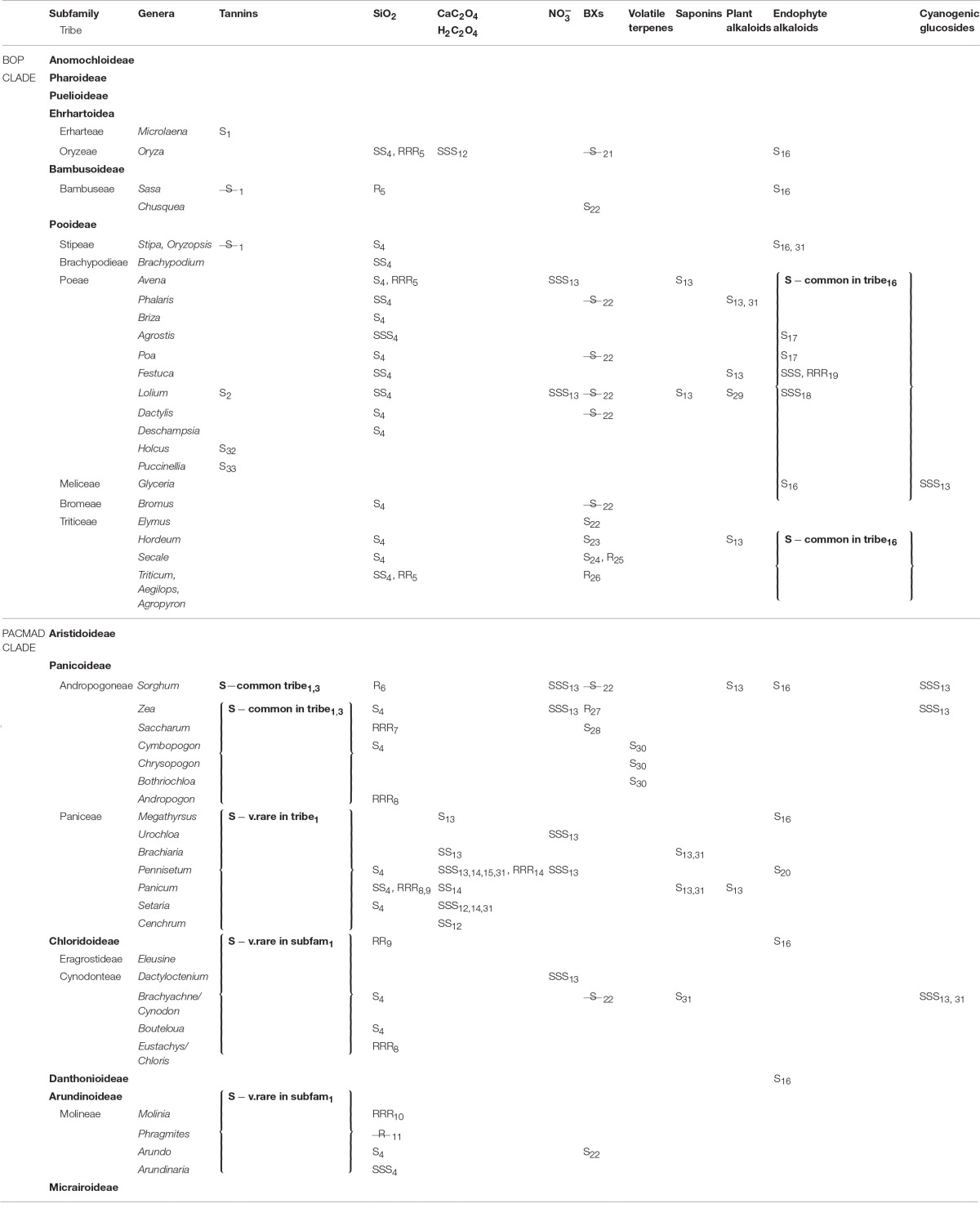
**Reported defenses in grass shoots and roots**.

*Table indicates the reported presence of tannins, silica (SiO_2_), oxalic acid, or calcium oxalate (H_2_C_2_O_4_, CaC_2_O_4_), nitrate (NO_3_−), benzoxazinoids (BXs), stored volatile terpenes such as monoterpenes, saponins, alkaloids of plant origin, alkaloids of endophytic fungal origin and cyanogenic glucosides. S, reported absence in shoots; S, reported presence in shoots (SS and SSS indicate more substantial concentrations); R, reported absence in roots; R, reported presence in roots (RR and RRR indicate more substantial concentrations)*.

*References: 1, Ellis, 1990; 2, Jackson et al., 1996; 3, Awika and Rooney, 2004; 4, Hodson et al., 2005; 5, Sangster, 1978; 6, Hodson and Sangster, 1989; 7, Parry and Kelso, 1977; 8,McNaughton et al., 1985; 9,Geis, 1978; 10, Parry and Kelso, 1975; 11, Schaller et al., 2013; 12, Rahman and Kawamura, 2011; 13, McKenzie, 2012; 14, Rahman et al., 2010; 15, Marais et al., 1997; 16, Clay, 1990; 17, Crawford et al., 2010; 18, Hennessy et al., 2016; 19, Patchett et al., 2008; 20, Ryley et al., 2007; 21, Kato-Noguchi and Peters, 2013; 22, Zuniga et al., 1983; 23, Barria et al., 1992; 24, Rice et al., 2005; 25, Copaja et al., 2006; 26, Lu et al., 2012; 27, Robert et al., 2012; 28, Singh et al., 2003; 29, Koulman et al., 2008; 30, Kaul and Vats, 1998; 31, Cheeke, 1995; 32, Iason et al., 1995; 33, Volz and Clausen, 2001*.

Regardless of where it is localized in roots, silica may contribute to the overall toughness of roots and might play a significant defense role in grasses where it is deposited in the root epidermis and throughout the root. Direct evidence that silica is involved in defense against grass root herbivores comes from a study by Frew et al. (2016b), which found that the relative consumption by and subsequent mass gain of root-feeding grayback canegrub (*D. albohirtum*) feeding on sugarcane roots was negatively correlated with silicon concentrations. It has also been suggested that silica in roots may also play an important role in resisting penetration by the huastoria of parasitic plants such as *Striga* (Hodson and Sangster, 1989).

### Calcium oxalate

Calcium oxalate (CaC_2_O_4_) is another mineral deposit that can serve as an inducible (Molano-Flores, 2001) anti-herbivore defense in many plant tissues, in addition to playing roles in structural support, Ca^2+^ regulation, protection against heavy metal toxicity, and drought tolerance (Franceschi and Nakata, 2005; Polley et al., 2013). In common with silica, calcium oxalate deposits are morphologically diverse, and include raphides, druses, and sands (Franceschi and Nakata, 2005). The deposits are hard and can abrade insect mouthparts (Korth et al., 2006) and reduce the digestibility of food by lepidopteran insects via a physical action (Park et al., 2009).

Crystalline calcium oxalate is reported from the shoots of many forage grasses from the large tribe Paniceae as well as from rice and bamboo, but may be absent from grasses more generally (Table 1, Libert and Franceschi, 1987; Cheeke, 1995; Prychid and Rudall, 1999; Rahman and Kawamura, 2011). Little published work has quantified calcium oxalate in grass roots, but Rahman et al. (2010) reported greater concentrations of soluble oxalate (discussed below) and similar concentrations of insoluble oxalate (calcium oxalate) in the shoots compared to the roots of *Pennisetum purpureum*. Raphides are concentrated in the root apical meristem in some other plants, apparently offering a defense against herbivores (e.g., Osuji, 2013). Hodson and Sangster (1989) observed concentrations of Ca in the outer cortical and epidermal cell walls of the subapical zone of *Sorghum* roots, and these might also be associated with calcium oxalate deposits. Some mycorrhizal fungal symbionts are also capable of synthesizing oxalic acid, which forms calcium oxalate in the presence of Ca^2+^ (Malajczuk and Cromack, 1982), and this might contribute to grass root defense due to its intimate association with the root.

## Get toxic—chemical defense

Grasses are less defended by toxic plant secondary metabolites (PSMs) or digestibility-reducing PSMs such as tannins than woody plants, but a variety of defenses have been cataloged from across the Poaceae, at least in the shoots (Table 1, Vicari and Bazely, 1993; Cheeke, 1995). Some defenses in grass roots are likely to be induced by herbivory, and although the oxylipin plant hormone, jasmonic acid (JA), is intimately involved in the initiation of induced defense responses, surprisingly little is known about the mechanisms of its action in grasses (Shyu and Brutnell, 2015). The limited evidence available suggests that the JA burst in response to attack, and the degree of localized induction, tend to be milder in plant roots than in shoots (Erb et al., 2012), although Erb et al. (2012) intriguingly postulate the existence of additional, unknown signals that may induce root defenses in the absence of a JA burst.

The toxicity of any PSM and its role in herbivore resistance is not absolute. Toxicity can be herbivore-specific and herbivore resistance is influenced by the intrinsic vulnerability and nutritional value of a plant tissue to herbivores, both negatively via compensatory feeding and positively via decreased palatability in different situations (Behmer, 2009; Erb et al., 2013; Johnson et al., 2014), as well as by the type and amount of PSMs and the interaction of nutrient and PSM levels (Behmer, 2009; Couture et al., 2016). Thus, the high fiber and low nutrient concentrations typical of most (but not all e.g., Tilman and Wedin, 1991) roots relative to leaves, may mean that herbivores can be deterred by a lower relative investment in chemical defense. Furthermore, the intrinsic chemical properties of some defense compounds makes them more effective in a below-ground environment than above-ground (van Dam et al., 2009), making direct comparisons between above- and below-ground levels of defense complicated. As is the case for above-ground herbivores (Bernays and Chapman, 1994), specialist root herbivores can also overcome chemical defenses and in some cases even use them as feeding cues (Johnson et al., 2011; Robert et al., 2012), however this does not preclude a role for these compounds in resistance against generalists.

### Benzoxazinoids

One well-studied chemical class almost entirely restricted to grasses (but occasionally reported from individual species from several dicotyledenous families (Adhikari et al., 2015) is the benzoxazinoids (BX), which includes the benzoxazolinone [e.g., benzoxazolin-2-one (BOA) and 6-methoxy-benzoxazolin-2-one (MBOA)], lactam [e.g., 2-hydroxy-1,4-benzoxazin-3-one (HBOA), 2-hydroxy-7-methoxy-1,4-benzoxazin-3-one (HMBOA)], and hydroxamic acid [e.g., 2,4-dihydroxy-1,4-benzoxazin-3-one (DIBOA), 2,4-dihydroxy-7-methoxy-1,4-benzoxazin-3-one (DIMBOA)] subclasses. These are widely reported from grass roots and root exudates, and appear to occur naturally both as glycosides and aglycones (Niemeyer, 2009), with the former stored in vacuoles in the roots (Copaja et al., 2006). Benzoxazinoids have been best studied for their roles in allelopathy and defense in the cereal crops maize, rye, and wheat, but also occur in many wild grasses (Zuniga et al., 1983). In root tissue, biosynthesis of BXs can be induced by competition (Rice et al., 2005; Lu et al., 2012) and by jasmonic acid or herbivory by *D. virgifera* (Robert et al., 2012). Allocation to roots relative to shoots can also increase in response to defoliation stress. Robert et al. (2012) show that allocation of DIMBOA to belowground parts of maize matches the predictions of optimal defense theory, with the greatest concentrations in the most nutritious crown roots. DIMBOA is deterrent to generalist root herbivores, and although several studies have reported positive correlations between BX concentrations and resistance to the specialist root herbivore *D. virgifera*, Robert et al. (2012) showed most recently that it is unaffected by DIMBOA and even uses high concentrations as a cue to locate its preferred (and most nutritious) crown roots. Nematodes appear to be relatively unaffected by BXs, with root knot nematodes *Meloidogyne incognita* in rye suppressed only at extremely high concentrations (Meyer et al., 2009) and reproduction of the stubby-root nematode *Paratrichodorus minor* unrelated to BX concentrations in maize. BXs are strongly involved in the resistance of some grasses to aboveground-feeding aphids, but this has not been demonstrated for root-feeding aphids (Niemeyer, 2009).

### Nitrate

Although a primary, rather than a secondary metabolite, excess nitrate in plant tissues presents a well-known risk of toxicity to grazing mammalian herbivores (Cheeke, 1998). Leaves of some grass genera, including *Sorghum, Avena, Lolium, Zea, Dactyloctenium*, and *Urochloa* can accumulate toxic nitrate levels in nitrogen-rich soils and after rain following dry periods, and this can be directly caustic to the gut lining (McKenzie, 2012). This is particularly damaging for monogastric vertebrates but might affect invertebrates as well. In vertebrates, nitrate toxicity is associated with the conversion of hemoglobin to methaemoglobin which cannot carry oxygen, and nitrate similarly reduces the affinity of haemocyanins for oxygen (Hazes et al., 1996; Cheng and Chen, 2002). The importance of these oxygen-binding proteins in insects is poorly understood (Hankeln et al., 2002), but may be underestimated, particularly for belowground herbivores. Nitrate is also a potent inhibitor of the midgut potassium pump in tobacco hornworm (*Manduca sexta*; Schirmanns and Zeiske, 1994) and its associated ATPase (Wieczorek et al., 1986). Although Hatcher et al. (1997b) showed that high nitrate levels in leaves of *Rumex obtusifolia* (Polygonaceae) were deterrent to chrysomelid beetles and Soucek and Dickinson (2012) demonstrated the toxicity of nitrate to aquatic insects, nitrate is not commonly considered as a plant defense. For now, data about root nitrate concentrations are scarce (Roumet et al., 2006), although substantial concentrations (>6% DM) are known to accumulate in the roots of wild-type *Arabidopsis* (Segonzac et al., 2007).

### Oxalic acid

Another primary metabolite involved in grass defense against herbivores is oxalic acid. The physical defense role of crystalline calcium oxalate has been discussed earlier, however much of the oxalate in grasses and particularly in roots may be in soluble form. Oxalate can inhibit feeding by homopteran insects (Yoshihara et al., 1980), and reduce larval growth rates in cotton bollworm (Yoshida et al., 1995). When free oxalic acid is consumed by herbivores, it can also form the insoluble salt, calcium oxalate, *in vivo—*potentially leading to nephrolithiasis (kidney stones) in both vertebrates and invertebrates (Hirata et al., 2012). This same process can reduce the bioavailablity of Ca^2+^, and oxalate thus acts as an antinutrient, potentially leading to hypocalcemia. In vertebrates, especially horses, this is a leading cause of “big head” syndrome (Cheeke, 1998) however any consequences for invertebrates are unknown.

High oxalate concentrations occur, usually along with calcium oxalate, in some tropical grasses and particularly in their roots (see above; also Rahman et al., 2010). Experimental evidence shows that oxalate (and calcium oxalate) synthesis increases in plants including spinach, rice and Napier grass (*Pennesetum purpureum*) with high availability of nitrate, but not of ammonium (Hatcher et al., 1997a; Rahman et al., 2010).

### Terpenoids

Only a small number of tropical aromatic grasses including *Cymbopogon, Bothriochloa, Vetiveria* and *Chrysopogon* (Kaul and Vats, 1998) possess specialized storage cells and accumulate significant concentrations of mono- and sesquiter penes in their leaves and stems (Lewinsohn et al., 1998). Evidence is currently lacking either for or against volatile terpene accumulation in grass roots. Other terpenoid products synthesized by grass roots but apparently not investigated as herbivore defenses include iridoid glycosides from maize (*Z. mays*) (Rengasamy et al., 2015) and diterpene momilactones from rice (*Oryza sativa*) (Kato-Noguchi and Peters, 2013).

Some grasses also produce steroidal saponins and sapogenins (saponin aglycones) that are derived from terpene precursors. Many of these are strongly molluscicidal (reviewed by Francis et al., 2002). Some triterpene saponins act against insects via their action as phytoecdysteroids, meaning that they mimic insect molting hormones. However, Dinan (1995) reported phytoecdysteroid activity in only five grass species (from the genera *Avena, Briza*, and *Festuca*) out of 45 tested, and then only from the seeds (the only part he tested). Although the genomes of these plants thus possess the capacity for phytoecdysteroid biosynthesis, this is not evidence of their presence in roots. Previously, similar activity had been reported from root extracts from the grass *Coix lachrymal-jobi* (Matsuoka et al., 1969 cited by Dinan, 1995), although more recent reports suggest that ecdysteroid activity of saponins may be attributable to increased membrane permeation rather than to direct effects on ecdysteroid receptors (De Geyter et al., 2012). While not universal, insect deterrence and toxicity have been observed for steroid and triterpene saponins from a variety of dicots, both above- and below-ground (Sutherland et al., 1982; De Geyter et al., 2010) although only antimicrobial actions have been reported for avenacins, triterpene saponins that accumulate in the roots of oats (Mylona et al., 2008).

Steroidal saponins, either alone or in synergy with other hepatotoxins, are associated with secondary (hepatogeneous) photosensitization in livestock feeding on many warm-climate grasses, including *Panicum* and *Cynodon* (Cheeke, 1995). These damage the liver, which is no longer able to remove the chlorophyll metabolite, phylloerythrin, to the bile for excretion. Sunlight then interacts with accumulated phylloerythrin, causing skin lesions, dermatitis, and photophobia. Invertebrate root herbivores are not exposed to sunlight or dietary chlorophyll, but may experience equivalent damage to systems for the elimination of toxic metabolites, such as ATP-binding cassette transporters (Robey et al., 2006).

### Alkaloids

Alkaloids are basic PSMs that contain nitrogen and are widespread plant defenses, both above- and below-ground. Endogenous alkaloids produced by grasses include hordenine, a phenylethylamine alkaloid from barley, sorghum, millet, and *Phalaris aquatica*. Hordenine is deterrent to *Heliothis* caterpillars (Bernays et al., 2000), grasshoppers (Harley and Thorsteinson, 1967), and ruminants (Marten et al., 1976). Indole alkaloids including gramine and perloline occur in barley, *P. aquatic*, and *Festuca arundinacea* (McKenzie, 2012). Gramine is toxic to aphids (Corcuera, 1984) and causes staggers and death in livestock (Binder et al., 2010). Perloline and hordenine are both most concentrated in roots, particularly soon after germination (Mann and Mudd, 1963; Gentry et al., 1969). Pyrrolizidine alkaloids were reported from a grass (*Lolium perenne*) for the first time relatively recently (Koulman et al., 2008).

Alkaloids of endophytic fungal origin present in grasses have been widely reviewed because of their detrimental effects on livestock (Clay, 1990; Saikkonen et al., 2013; Schardl et al., 2013), although some specifically affect insects, including root herbivores (Popay et al., 2004; Hennessy et al., 2016). They include lolines, peramine, ergot alkaloids, and indole diterpenes including epoxy-janthitrems produced by endophytes from the genera *Epichloë* and *Neotyphodium*, most prominently in the grass genera *Festuca* and *Lolium*, but also in native North American grasses (Crawford et al., 2010) and numerous cool-climate southern hemisphere grasses (Moon et al., 2002, 2007). However, the conferral of herbivore resistance by endophytes of native grasses is generally weaker and less consistent than in agronomic grasses (Faeth and Fagan, 2002). Although endophytes and their alkaloids are usually not detected when grass roots are analyzed (Clay, 1990; Elmi et al., 2000) endophyte infection appears to be highly detrimental to root knot nematodes of *Festuca* (Elmi et al., 2000) and to deter the root herbivore *Costelytra zealandica* (Rostás et al., 2015), although deterrence in the latter case might also be explained by altered volatile emissions. Evidence that endophyte infection is detrimental to sap-feeding aphids (Wilkinson et al., 2000; Popay et al., 2004) is consistent with observations that lolines can be transported in phloem to grass roots (Burhan, 1984; Patchett et al., 2008; Omacini et al., 2012).

Other grass endophytes may also produce steroidal toxins such as wortmannin, which may be responsible for kikuyu staggers in cattle (Ryley et al., 2007), however, their relevance to root defense and to invertebrate herbivores is unknown.

### Amino acids

Non-proteinogenic amino acids can be toxic and sometimes afford effective defense. *M*-tyrosine can reach concentrations of up to 43% of root exudate dry matter in some *Festuca* species, is allelopathic (Bertin et al., 2007) and can reduce cabbage looper (*Trichoplusia ni*) growth rates when expressed in *Arabidopsis thaliana* (Huang, 2010). Another tyrosine isomer, β-tyrosine, is inducible, and abundant in the roots and root exudates of some rice cultivars, but despite also being strongly allelopathic has no detectable effects on hemipteran or lepidopteran herbivores in bioassays (Yan et al., 2015).

### Cyanogenic glucosides

In common with most plant families, some grasses e.g., *Brachyachne, Cynodon, Glyceria, Zea*, and *Sorghum*, (particularly Johnson grass, *S. halapense* and Sudan grass, *S*. × *drummondii*) produce cyanogenic glucosides including limarin and dhurrin, and these too have been well-studied in some species because they impact livestock (Cheeke, 1998; McKenzie, 2012). Cyanogenic glucosides are deterrent to most generalist insects (Gleadow and Woodrow, 2002) but can be tolerated or avoided by some specialists (Engler et al., 2000). Cyanogenic glucosides are present in roots of *Cynodon dactylon* where an allelopathic role has been proposed (Mahmoodzadeh, 2010) and provide nematocidal benefits in the root epidermis of several *Sorghum* species (Curto et al., 2012). Cyanogenic glucoside concentrations are generally highest in young plants and plants exposed to drought or N-rich soils (Gleadow et al., 2016).

### Tannins and other phenolics

Tannins are large polyphenolic compounds found widely in shoots and roots generally and best-known for their ability to form insoluble (and thus indigestible to vertebrates) complexes with dietary protein. It should be noted that for most insects, the potential pro-oxidative activity of tannins is more biologically important than protein-precipitating effects, which do not occur in typically-alkaline insect guts (Appel, 1993; Salminen and Karonen, 2011). Tannins are very rare and/or in extremely low concentrations in grasses, although some grasses, including sorghum, barley, rice, wheat, red finger millet (*Eleusine coracana*), *Festuca arundinacea* and *Lolium perenne*, produce condensed tannins in their caryopsis (grain) (McCallum and Walker, 1990; Gu et al., 2003; Awika and Rooney, 2004; Dykes and Rooney, 2007; Fraser et al., 2016). To our knowledge, grass roots have not been investigated for tannins.

Based on a histological survey, Ellis (1990) and Chesselet et al. (1992) reported “tannin-like substances” from the leaf epidermis of 39 genera (of 1,104 species in 290 genera inspected) of South African grasses, mostly in C4 species growing in poor soils, suggesting that tannins may occur more widely among grasses than is commonly recognized. The presence in leaves of ellagitannins in the arctic grass *Puccinellia artica* (Volz and Clausen, 2001) and condensed tannins in *Holcus lanatus* (Iason et al., 1995) and in *Lolium perenne* and *Digiteria sanguinalis* (Jackson et al., 1996) has also been reported on the basis of colorimetric tests, however more recent LC-MS analysis failed to detect tannins in *L. perenne* and *F. arundinace* leaves (Fraser et al., 2016). In all cases, tannin concentrations in grass vegetative tissue are low and seem unlikely to afford substantial herbivore resistance. Only weak correlative links have been presented between grass tannins and herbivore feeding preferences (Capinera et al., 1983; Volz and Clausen, 2001), while Mole and Joern (1994) concluded that condensed tannins were ineffective against grasshoppers. Simple monomeric phenolics have been little investigated in grass roots, although chlorogenic acid has been implicated in the resistance of maize roots to herbivory (Nuessly et al., 2007; Robert et al., 2012).

Phenolic compounds including tannins, phenolic acids, flavonoids, and anthocyanins are almost ubiquitous in plants, but whether they play any role in species-species interactions is unpredictable and highly structure-dependent (Lane et al., 1985; Barbehenn and Constabel, 2011; Moore et al., 2014). The absolute quantification of “total phenolics” using standard assays is highly problematic (Appel et al., 2001) and without detailed compound identification, useful conclusions about biological activity are not possible. For example, Parker et al. (2012) detected similar concentrations of “total phenolics” in roots and shoots of *Oenothera biennis*, but the allocation of particular phenolics, with differing biological activities, to roots vs. shoots differed.

In recent years, the capacity to identify and quantify individual phenolics, phenolic glycosides and polyphenolics including ellagitannins, has improved dramatically (Salminen et al., 2011) and lead to valuable insights into the role of phenolics in plant defense (e.g., Agrawal et al., 2012). Another useful approach is to implement assays that estimate putative chemical mechanisms of these compounds, such as oxidative activity measured at a pH comparable to the midgut lumen pH of insects (Barbehenn et al., 2006; Salminen and Karonen, 2011). These detailed chemical and mechanistic approaches offer the most promising way forward but have yet to be applied to grass roots.

### Proteinase inhibitors

Proteinase inhibitors (PI) are important defenses against herbivory in many plants, including Solanaceous and Leguminous crops, where they harm herbivores by inhibiting the action of digestive proteinases such as trypsin and chymotrypsin (Farmer, 2014). Most published examples concern aboveground herbivory and relatively few reports exist from grasses, although the presence and induction by JA of subtilisin/chymotrypsin-inhibiting-type PIs have been reported from the leaves of *Brachypodium distachyon* and from wheat endosperm (Mur et al., 2004; Tedeschi et al., 2012). A cysteine protease from maize, which is expressed in all tissues, has been shown to serve in insect resistance by damaging to the peritrophic matrix of lepidopteran larvae (Pechan et al., 2002), and the gene encoding it is also highly expressed in sugarcane roots (Falco et al., 2001). Root herbivory by the southern corn rootworm and western corn rootworm and by experimental application of JA induces the transcription of proteinase inhibitor genes in maize roots (Lawrence et al., 2012; Robert et al., 2012).

## Get a bodyguard—recruitment of natural enemies

All grasses tested to date have been shown to emit volatiles following aboveground herbivory (Degenhardt, 2009) and these often recruit the natural enemies of herbivores, but the situation belowground is relatively less explored. Maize releases a sesquiterpene, (E)-β-caryophyllene, from its roots following attack by the western corn rootworm, attracting entomopathogenic nematodes (EPNs, Rasmann et al., 2005). EPNs infect the bodies of root-feeding insects and cause septicaemia by releasing bacteria while reproducing in the dying insect. Similarly, northern white cedar (*Thuja occidentalis*), and *Citrus* release chemical signals (a C_12_ terpene in the citrus) that attract entomopathogenic nematodes when their roots are attacked by weevils (*Otiorhynchus sulcatus* and *Diaprepes abbreviatus*, van Tol et al., 2001; Ali et al., 2010) and roots of *Panicum bisulcatum* treated with JA (inducing a defensive response) attracted EPNs in soil olfactometers, suggesting this grass species was emitting VOCs that recruited these natural enemies of root herbivores (Hiltpold et al., 2016). The induced emission of volatile terpenes by the grasses *F. arundinacea* and *Poa pratensis* following root herbivory by beetles attracts *Tiphia* parasitoid wasps from aboveground, that subsequently burrow into the soil to attack the grubs (Obeysekara et al., 2014). There is therefore good reason to believe these mechanisms are just as common belowground as aboveground (Turlings et al., 2012).

Microbes and mycorrhizal fungi that interact with grass roots might also play roles as bodyguards, or otherwise alter plant-herbivore interactions. The plant parasitic nematode *Tylenchorhynchus ventralis* is strongly controlled by soil microbes in a coastal foredune grassland (Piskiewicz et al., 2009) and colonization of maize by the rhizobacterium *Azospirillum brasilense* can deter, and reduce the performance of, western corn rootworm (Santos et al., 2014). However, another study of three plant species, including the grass *Holcus lanatus*, found no effect of the soil microbial community on defense against nematodes (Wurst et al., 2009). Alkaloid-producing endophytes were discussed above, and can also be considered to be bodyguards. However, endophyte infection has also been shown to alter volatile emissions from grass roots (Rostás et al., 2015), and may interfere with the recruitment of natural enemies.

## Conclusions

Most of the grass defenses described above are known from limited sections of the Poaceae, and primarily from crop and pasture species. While economically and often ecologically important, these grass species are not representative of the phylogenetic, morphological, and ecological diversity present in this large and cosmopolitan plant family. Most of these species have been subject to artificial selection through the process of domestication, and this process can alter plant-herbivore interactions and often cause the diminution or loss of plant defenses (Rosenthal and Dirzo, 1997; Kollner et al., 2008; Turcotte et al., 2014; Chen et al., 2015). Furthermore, many crop species experience very little above-ground herbivory by mammalian grazers and may consequently differ from wild grasses that have evolved alongside ungulate herbivores and which may differ in their relative reliance on the resistance and tolerance elements of defense. It has been suggested that grasslands supporting populations of large grazing vertebrates such as ungulates and macropods are more tolerant of grazing than ungrazed grasslands (Rosenthal and Kotanen, 1994). However, tolerance traits such as protected meristems, compensatory growth and compensatory photosynthesis may sometimes be adaptations to fire and drought, rather than, or as well as, adaptations to herbivory (Rosenthal and Kotanen, 1994).

These observations highlight the need for systematic surveys of defense throughout the family. As well as shining a light on phylogenetic patterns of defense, this approach may enable the identification of defense syndromes and/or defense tradeoffs where they exist. Plant defense theories offer many predictions about differential patterns of defense and herbivory between C_3_ and C_4_ plants; between domesticated and wild plants; throughout ecological succession (Rasmann et al., 2011) and along environmental gradients of temperature, precipitation, soil and fire frequency; yet these predictions remain largely untested in grasses, let alone below-ground.

Given the size and diversity of the Poaceae, there are likely many undiscovered and unexplored chemical defenses and defense strategies to be found belowground. In particular, there may be much to learn from studies of allelopathic secondary metabolites produced by plant roots. Numerous examples exist of phytotoxic compounds synthesized and exuded by grass roots, which have not been tested for roles against root herbivores, or at least, such tests have not been reported. Examples include diterpene momilactones produced by the roots of rice, which are induced by jasmonic acid (Kato-Noguchi and Peters, 2013), antimicrobial triterpene saponins known as avenacins which accumulate in oat roots (Mylona et al., 2008), and sorgoleone, a hydrophobic ρ-benzoquinone exuded from Sorghum root hairs (Weston et al., 2013). In surveying the relevant literature, we noted an apparent lack of consideration given to the possibility that grass rhizosheaths may play a role in defense against herbivores. We also identified a need to characterize the contribution of root composition (in terms of cellulose, lignin, callose, suberin, silica, and calcium oxalate) to root toughness, and the significance of root toughness for defense against herbivores. More broadly, we hope that in surveying the relevant literature, we have equipped researchers with candidate grass root defenses for further hypothesis-driven research.

## Author contributions

BM and SJ conceived the review article. BM reviewed the literature and wrote the paper with significant input from SJ.

## Funding

The work was funded by a Discovery grant (DP140100363) from the Australian Research Council to the authors.

### Conflict of interest statement

The authors declare that the research was conducted in the absence of any commercial or financial relationships that could be construed as a potential conflict of interest.
